# ICD-11: Änderungen der diagnostischen Kriterien der Substanzabhängigkeit

**DOI:** 10.1007/s00115-021-01071-7

**Published:** 2021-02-16

**Authors:** Andreas Heinz, Melissa Gül Halil, Stefan Gutwinski, Anne Beck, Shuyan Liu

**Affiliations:** 1 Klinik für Psychiatrie und Psychotherapie, Charité Universitätsmedizin Berlin, Campus Bonhoefferweg 3, Charitéplatz 1, 10117 Berlin, Deutschland; 2 Psychiatrische Universitätsklinik der Charité im St. Hedwig Krankenhaus, Große Hamburger Str. 5–11, 10115 Berlin, Deutschland

**Keywords:** Alkoholkonsumstörung, Diagnose, Klassifikation, ICD-11, ICD-10, Alcohol use disorder, Diagnosis, Classification, ICD-11, ICD-10

## Abstract

**Hintergrund:**

In der ICD(International Classification of Diseases)-11 ändern sich die Kriterien für die Diagnose der Substanzabhängigkeit.

**Ziel der Arbeit (Fragestellung):**

Erörterung der Vor- und Nachteile der neu verfassten Diagnosekriterien.

**Material und Methoden:**

Diskussion unter Berücksichtigung neurobiologischer, sozialer und klinischer Forschungsergebnisse.

**Ergebnisse:**

Im ICD-11 werden wie bisher Abhängigkeitserkrankungen und schädlicher Gebrauch unterschieden. Zur Diagnose der Abhängigkeit werden die ehemals 6 Diagnosekriterien in 3 Paaren gebündelt, von denen künftig 2 erfüllt sein müssen. Innerhalb der Paare genügt ein erfülltes Kriterium, damit das Paar als bejaht gilt. Unter Bezugnahme wissenschaftlicher Erkenntnisse im Suchtbereich zeigen sich Vor- und Nachteile. Hierbei könnte sich die Spezifität der Diagnosestellung gegenüber dem ICD-10 verschlechtern, da pro Paar nur ein Kriterium erfüllt sein muss und somit die Möglichkeit besteht, dass nicht problematisches Konsumverhalten inkorrekt pathologisiert und falsch diagnostiziert wird. Das erscheint als problematisch, da die Definition des ICD-10 „Anhaltender Konsum trotz eindeutiger schädlicher Folgen“, im ICD-11 weiter gefasst wird als „Oft fortgeführter Konsum trotz Auftreten von Problemen“. Dies könnte dazu führen, dass das Kriterium einfach deshalb erfüllt wird, weil der Konsum einer Substanz in einem bestimmten Land illegal ist. In der bisher größten multinationalen Studie in 10 Ländern zur Konkordanz der Diagnosesysteme wurde unter Verwendung der ICD-11 die Alkoholabhängigkeit ca. 10 % häufiger gestellt als mittels ICD-10.

**Schlussfolgerung:**

In der ICD-11 werden die Diagnosen der Substanzabhängig und des Missbrauchs als klinisch sinnvolle Syndrome aufrechterhalten. Ob die diagnostischen Neuerungen in der Praxis hilfreich sind oder beispielsweise negative soziale Auswirkungen für die Betroffenen im Sinne unangemessener Pathologisierung mit sich bringen, ist systematisch zu prüfen.

## Abstract (en)

**Background:**

In ICD(International Classification of Diseases)-11 the criteria for the diagnosis of substance dependence have been revised.

**Objective:**

Discussion of the advantages and disadvantages of the revised diagnostic criteria.

**Material and methods:**

Discussion of criteria with respect to neurobiological, social and clinical research.

**Results:**

In the new ICD-11, harmful drug use and substance dependence remain separate diagnostic categories. Regarding substance dependence, the former six diagnostic criteria are condensed into three pairs, two of which must be fulfilled to diagnose this disorder. Within the pairs, one affirmed symptom or aspect is sufficient for the new criterion to be fulfilled. With reference to scientific findings in the field of addiction, this appears to provide advantages as well as disadvantages. The specificity of the diagnosis of substance dependence might get worse compared to ICD-10, because only one symptom of the syndrome pairs has to be fulfilled in each newly constructed pair in order to fulfill the criterion. Therefore, the risk of false positive diagnosis of substance dependence could increase.

This is a potential reason for concern because the definition of ICD-10 “persisting substance use despite clear evidence of overtly harmful consequences”, is more broadly reworded in ICD-11 as “substance use often continues despite the occurrence of problems”. This criterion may hence simply be fulfilled because a substance is illegal in a certain country. In the largest multinational study in 10 countries concerning concordance of diagnostic systems, alcohol dependence was diagnosed approximately 10% more often using ICD-11 compared to ICD-10.

**Conclusion:**

In ICD-11 the well-established distinction between substance dependence and harmful drug use is preserved. Systematic studies are required to assess whether the new diagnostic criteria are clinically helpful or increase social problems by increasing false positive diagnoses of addiction.

## Hinführung zum Thema

Mit der 11. Version des International Statistical Classification of Diseases and Related Health Problems (ICD-11) werden die Kriterien für die Diagnose einer Substanzabhängigkeit neu formuliert. Neben der Veröffentlichung der Beschreibung der Substanzabhängigkeit durch die World Health Organization (WHO; Tab. 1; [1]) wurde von Mitgliedern der Arbeitsgruppe der WHO zur Erstellung des Kapitels „Mental, Behavioural or Neurodevelopmental Disorders“ des ICD-11 ein Entwurf der diagnostischen Kriterien der Substanzabhängigkeit veröffentlicht, auf die sich unsere Diskussion bezieht (Tab. 2; [2]). Die endgültigen diagnostischen Kriterien (CDDG, Clinical Description and Diagnostic Guidelines) werden voraussichtlich im Verlauf von 2021 veröffentlicht. Zudem verfolgt die WHO erstmalig einen offenen Ansatz, sodass es neben einer festen Version („frozen release“) auch eine offen Version („maintenance platform“) geben wird, in der Änderungen eingepflegt werden können und die dann in regelmäßigen Abständen in die „frozen version“ überführt wird.

**Table 1:** 

ICD-11 Abhängigkeit	ICD-10 Abhängigkeitssyndrom
Eine Störung der Regulierung von Substanzgebrauch, die durch wiederholten oder kontinuierlichen Konsum entsteht Charakteristisches Merkmal ist ein starkes Verlangen, die Substanz zu konsumieren, welches sich durch die fehlende Fähigkeit manifestiert, den Konsums zu kontrollieren, einer zunehmenden Priorisierung des Konsums gegenüber anderen Aktivitäten und fortgeführten Konsum trotz Schädigung oder negativer Konsequenzen. Diese Erfahrung ist häufig begleitet durch subjektives Verlangen oder Drang zu konsumieren. Physiologische Merkmale der Abhängigkeit können ebenfalls bestehen, einschließlich Toleranz gegenüber der Substanz, Auftreten von Entzugssymptomen nach Absetzen oder Reduktion der Substanz oder Konsum einer gleichartigen Substanz, um Entzugssymptome zu verhindern oder abzuschwächen. Die Merkmale der Abhängigkeit bestehen in der Regel in einem Zeitraum von 12 Monaten, oder die Diagnose kann auch bei Substanzkonsum bei anhaltendem (täglich oder fast täglich) gestellt werden	Eine Gruppe von Verhaltens-, kognitiven und körperlichen Phänomenen, die sich nach wiederholtem Substanzgebrauch entwickeln. Typischerweise besteht ein starker Wunsch, die Substanz einzunehmen, eine verminderte Kontrolle über ihren Konsum und anhaltender Substanzgebrauch trotz schädlicher Folgen. Dem Substanzgebrauch wird Vorrang vor anderen Aktivitäten und Verpflichtungen gegeben. Es entwickelt sich eine Toleranzerhöhung und manchmal ein körperliches Entzugssyndrom Das Abhängigkeitssyndrom kann sich auf einen einzelnen Stoff beziehen (z. B. Tabak, Alkohol oder Diazepam), auf eine Substanzgruppe (z. B. opiatähnliche Substanzen) oder auch auf ein weites Spektrum pharmakologisch unterschiedlicher Substanzen

Die Übersetzung des ICD-11 ist keine offizielle Übersetzung, sondern erfolgte durch die Autoren (Beschreibung des ICD-11 [„Description“] nach der WHO [1], aktuelle „frozen Version“, ICD-10 nach Freyberger und Dillinger [3])

*ICD* International Classification of Diseases, *WHO* World Health Organization

**Table 2:** 

ICD-11 Abhängigkeit	ICD-10 Abhängigkeitssyndrom
Die Diagnose erfordert, dass 2 oder mehr der 3 zentralen Kriterien über einen Zeitraum von mindestens 12 Monaten bestehen, kann aber auch gestellt werden, wenn die Substanz mindestens einen Monat kontinuierlich (täglich oder fast täglich) konsumiert wird	Um die Diagnose eines Abhängigkeitssyndroms stellen zu können, müssen 3 oder mehr Kriterien mindestens einen Monat lang gleichzeitig oder wiederholt innerhalb von 12 Monaten vorhanden sein
1 Beeinträchtigte Kontrolle über den Substanzkonsum – Bezogen auf Beginn, Menge und Umstände oder Ende des Konsums. Wird oft, aber nicht notwendigerweise von subjektiven Empfindungen von Drang oder Verlangen, die Substanz zu konsumieren, begleitet	1 Ein starkes Verlangen („craving“) oder eine Art Zwang, die Substanzen zu konsumieren
	2 Verminderte Kontrolle über den Substanzgebrauch, d. h. über Beginn, Beendigung oder die Menge des Konsums, deutlich daran, dass oft mehr von der Substanz oder über einen längeren Zeitraum konsumiert wird als geplant, oder an dem anhaltenden Wunsch oder an erfolglosen Versuchen, den Substanzkonsum zu verringern oder zu kontrollieren
2 Physiologische Merkmale (indikativ für substanzbezogene Neuroadaption) manifestiert sich als: (i) Toleranz, (ii) Entzugserscheinungen nach Konsumstopp oder -reduktion oder (iii) wiederholter Konsum der Substanz, um Entzugserscheinungen zu mindern oder zu verhindern Entzugserscheinungen müssen dem Entzugssyndrom der Substanz entsprechen und sind nicht auf anhaltende Substanzeffekte zurückzuführen	3 Toleranzentwicklung gegenüber den Wirkungen der Substanz. Für eine Intoxikation oder um den gewünschten Effekt zu erreichen, müssen größere Mengen der Substanz konsumiert werden, oder es treten bei fortgesetztem Konsum derselben Menge deutlich geringere Effekte auf
	4 Ein körperliches Entzugssyndrom, wenn die Substanz reduziert oder abgesetzt wird, mit den für die Substanz typischen Entzugssymptomen oder auch nachweisbar durch den Gebrauch derselben oder einer sehr ähnlichen Substanz, um Entzugssymptome zu mildern oder zu vermeiden
3 Substanzkonsum wird fortschreitend zur Priorität im Leben, d. B., dass die Substanz Vorrang über andere Interessen, Vergnügungen, alltägliche Aktivitäten, Verpflichtungen oder der Gesundheitspflege oder persönlichen Pflege erhält. Der Substanzkonsum nimmt zunehmend eine zentrale Rolle im Leben der Person ein und verschiebt andere Aspekte des Lebens in die Peripherie und wird oft trotz des Auftretens von Problemen fortgeführt	5 Einengung auf den Substanzgebrauch, deutlich an der Aufgabe oder Vernachlässigung anderer wichtiger Vergnügungen oder Interessensbereiche wegen des Substanzgebrauchs; oder es wird viel Zeit darauf verwandt, die Substanz zu bekommen, zu konsumieren oder sich davon zu erholen
	6 Anhaltender Konsum trotz eindeutiger schädlicher Folgen, deutlich an dem fortgesetzten Gebrauch, obwohl der Betreffende sich über Art und Ausmaß des Schadens bewusst ist oder bewusst sein könnte

Die Übersetzung des ICD-11 ist keine offizielle Übersetzung, sondern erfolgte durch die Autoren (Diagnostische Kriterien nach Saunders et al. [2]); ICD-10 nach Dillinger und Freyberger [3]

*ICD* International Classification of Diseases

Anders als im Diagnostic and Statistical Manual of Mental Disorders (DSM-5) [4] werden in der ICD-11 die diagnostischen Kategorien für den schädlichen Gebrauch und die Abhängigkeitserkrankung des ICD-10 aufrechterhalten. Wir diskutieren Vor- und Nachteile der aktuellen Neuerungen vor dem Hintergrund neurobiologischer, sozialer und klinischer Befunde.

## Hintergrund

In der ICD-11 werden gegenüber der ICD-10 die Kriterien für die Diagnose einer Substanzabhängigkeit neu definiert ([2, 3, 5]; Überblick siehe Tab. 2). Abhängigkeitserkrankungen werden weiterhin vom schädlichen Gebrauch einer Substanz unterschieden. Der Nachteil eines solchen Vorgehens ist, dass der dimensionale Charakter der Substanzgebrauchsstörungen damit nicht gut abgebildet wird. Das heißt der häufig gleitende Übergang vom exzessiven, genussorientierten Substanzgebrauch zu habituellem Drogenkonsum, der schließlich sogar trotz schwerwiegender persönlicher Nachteile fortgesetzt wird, wird weiterhin kategorial in unterschiedliche Störungsbilder unterteilt. Die Beibehaltung der Unterscheidung zwischen dem schädlichen Substanzgebrauch und dem abhängigen Drogenkonsum hat aber den Vorteil, dass das relativ klar definierte Krankheitsbild der Abhängigkeitsstörung nicht wie im DSM‑5 mit der Kategorie des schädlichen Konsums vermengt wird, die in viel stärkerem Ausmaß durch soziale Wertungen und Regulierungen beeinflussbar ist [6].

Ob der Konsum einer Substanz für die betroffene Person mit negativen Konsequenzen verbunden ist, liegt nicht nur an den direkten Auswirkungen der Droge selbst, sondern auch an sozialen Faktoren wie etwa der Frage, ob Erwerb und Konsum der Droge im jeweiligen Land legal oder illegal sind. Die Beeinträchtigung wesentlicher Rollenerwartungen, das Auftreten negativer Konsequenzen etwa beim Versuch, eine illegale Droge zu beschaffen, und Auseinandersetzungen im persönlichen Umfeld auch beim noch nicht abhängigen Konsum einer Droge werden somit von den gesellschaftlichen Rahmenbedingungen entscheidend beeinflusst [7]. Wer versucht hat, ein Glas Wein in einem Land zu konsumieren, in dem dies gesetzlich verboten ist, kann diese Erfahrung auch als deutsche Staatsbürgerin oder Staatsbürger machen. Die wesentlichen Veränderungen der vorab veröffentlichten Kriterien der ICD-11 liegen demgegenüber in der Neugruppierung der Symptome einer Abhängigkeitserkrankung. Dieser Entwurf der Kriterien soll im Folgenden diskutiert und mit dem Stand der Kenntnisse bez. neurobiologisch und klinisch gut belegter Mechanismen der Entwicklung und Aufrechterhaltung einer Abhängigkeitserkrankung verglichen werden. In der ICD-11 werden die bisherigen 6 Kriterien der Abhängigkeitserkrankung zu 3 Paaren zusammengefasst, die jeweils 2 der bisherigen Kriterien als Aspekte des neuen gemeinsamen Kriteriums auflisten. Von diesen 3 neuen Kriterien müssen 2 erfüllt sein, um eine Abhängigkeitserkrankung diagnostizieren zu können, bisher waren es 3 von 6 in der ICD-10 [8]. Möglicherweise wird die genaue Formulierung der Kriterien noch verändert, in der vorliegenden Form könnte aber die Schwelle zur Diagnosestellung herabgesetzt werden, da in jedem der 3 neuen „Doppelkriterien“ nur Aspekte der (früher unabhängig voneinander bewerteten) Kriterien erfüllt sein müssen. So heißt es beispielsweise bei ICD-11-Kriterium 1, dass der Kontrollverlust „oft, aber nicht notwendigerweise von subjektiven Empfinden oder Drang“ begleitet wird. Bei Kriterium 2 können die früher als eigenständige Kriterien separat gelisteten Aspekte gemeinsam vorkommen, müssen es aber nicht, denn es ist die Rede vom Auftreten von Toleranzentwicklung, Entzugserscheinungen *oder *anhaltenden Konsum zur Verhinderung von Entzugssymptomen [2]. In Kriterium 3 steht die Priorität im Vordergrund, die dem Substanzkonsum im Leben der Betroffenen eingeräumt wird, die Fortführung des Substanzkonsums trotz negativer Konsequenzen trete „oft“ hinzu, ist aber zur Erfüllung des Kriteriums nicht notwendig [2].

Da die *Clinical Description and Diagnostic Guidelines* der WHO noch nicht offiziell veröffentlicht sind, ist es möglich, dass sich die Zusammensetzung der Kriterien oder Inhalte im Verlauf noch ändern werden. Die vorliegende Arbeit bezieht sich auf den vorab veröffentlichten Entwurf der Kriterien des ICD-11 von Saunders und Kollegen [2], die Mitglieder der WHO-Arbeitsgruppe sind.

## Toleranzentwicklung und Entzugssymptomatik

Schon lange wurde argumentiert, dass die Entzugssymptomatik nur ein Aspekt der vorherigen Toleranzentwicklung ist, da es sich bei beiden Symptomen neurobiologisch um die Folge neuroadaptive Veränderungen handelt, die im Sinne einer Aufrechterhaltung der Homöostase der Drogenwirkung entgegengesetzt sind [9, 10]. Wirkt also beispielsweise Alkohol GABAerg und damit inhibierend und subjektiv sedierend und hemmt Alkohol zudem die glutamaterge Neurotransmission am NMDA-Rezeptor, dann werden entsprechend bei chronischem Alkoholkonsum GABAerge Rezeptoren in ihrer Sensitivität herabreguliert, während NMDA-Rezeptoren hochreguliert werden [11, 12]. Damit kann unter der Voraussetzung des fortgesetzten Alkoholkonsums eine neue Homöostase erreicht werden, d. h. die dämpfende und inhibierende Wirkung des Alkohols wird ausgeglichen durch eine entsprechende Neuroadaptation der inhibitorischen und exzitatorischen Systeme im Gehirn. Fällt der Alkoholkonsum plötzlich weg, was bei schwer abhängigen Menschen bereits durch die Unterbrechung des Trinkens während des Schlafes geschehen kann, dann ist die inhibitorische Wirkung der GABAergen Neurotransmission reduziert, während eine erhöhte Zahl der NMDA-Rezeptoren zur zentralnervösen Erregung beitragen kann. Insofern hier auch die Regulation zentralnervöser Kerngebiete betroffen ist, die das autonome Nervensystem steuern (wie beispielsweise der Locus coeruleus als Ursprungsgebiet noradrenerger Neurone im Hirnstamm), kann es zur vegetativen Dysfunktion und damit zu den auch körperlich sichtbaren Zeichen eines Entzugs kommen [13]. Deshalb ist es sinnvoll, Toleranzentwicklung und Entzugssymptomatik als gemeinsames Geschehen zu verstehen und die beiden bisher getrennt gelisteten Leitkriterien als zwei Aspekte eines gemeinsamen Kriteriums im ICD-11 zusammenzufassen.

Das Kriterium der Toleranzentwicklung wird seit längerer Zeit kritisch diskutiert, da die Toleranzentwicklung im Rahmen der klinischen Untersuchungen meist anamnestisch, retrospektiv erhoben wird und die Erinnerung täuschen kann. Es kommt hinzu, dass etwa Toleranz gegen die akuten Alkoholwirkungen partiell genetisch bedingt ist [14]. Dementsprechend können Menschen, die bereits während der ersten Erfahrungen mit der Substanz Alkohol wenig unerwünschte Wirkungen wie Sedation oder Übelkeit zeigen, häufiger zu einem erhöhten Alkoholkonsum neigen, wahrscheinlich, weil ihnen entsprechende Warnzeichen fehlen [15]. Im Nachhinein ist allerdings schwer zu unterscheiden, was bereits angeborene oder möglicherweise auch stressbedingt früh erworbene Toleranz gegenüber den akuten Alkoholwirkungen ist und was sich erst in Folge fortgesetzten Alkoholkonsums als Toleranzentwicklung gegenüber der Drogenwirkung eingestellt hat. Deshalb erscheint es sinnvoll, das alleinige Auftreten einer Entzugssymptomatik als hinreichend zu werten, um das Kriterium als erfüllt zu betrachten.

Die Erfüllung eines dieser neuen Kriterien reicht allerdings nicht aus, um eine Abhängigkeitserkrankung zu diagnostizieren. Das ist sehr berechtigt, da sich Entzugssymptome auch bei anderen zentralnervös wirksamen Drogen und Medikamenten finden. Letztendlich sind neuroadaptive Anpassungen an die Substanzwirkung ubiquitär und finden sich auch bei Einnahme von ß‑Blockern, Antidepressiva oder anderen Medikamenten, die keine Abhängigkeitserkrankung verursachen [16]. Um eine Suchterkrankung bzw. eine Abhängigkeitserkrankung in der Terminologie der ICD-10 und -11 zu diagnostizieren, ist demnach auch weiterhin das Erfüllen zusätzlicher Kriterien notwendig.

## Craving und verminderte Kontrolle über den Substanzgebrauch

Auch die Symptome des starken Drogenverlangens („craving“) und der verminderten Kontrolle über den Substanzgebrauch werden in der ICD-11 gemeinsam gelistet und es genügt, dass einer dieser beiden Aspekte vorliegt, damit das Kriterium erfüllt ist. Auch diese Gruppierung erscheint neurobiologisch und klinisch sinnvoll, da die Kontrolle über den Substanzmittelgebrauch aus unterschiedlichen Gründen reduziert oder ganz verloren sein kann. Ein wesentlicher Grund hierfür ist das starke Verlangen, das betroffene Personen dazu bringt, ihre bewusst gefassten Vorsätze nach Reduktion des Suchtmittelkonsums oder nach Abstinenz nicht einzuhalten. Die neurobiologisch orientierte Suchtforschung hat in den letzten Jahrzehnten eine Vielzahl zentralnervöser Korrelate des Suchtverlangens und der Manifestation impulsiv oder zwanghaft anmutender Verhaltensweisen identifizieren können [3, 8, 17]. Schon lange ist bekannt, dass Substanzen mit Abhängigkeitspotenzial deutlich mehr Dopamin im Bereich des ventralen Striatums, einer Kernregion des sog. hirneigenen Belohnungssystems, freisetzen und dass diese Freisetzung auch bei wiederholtem Drogenkonsum nicht abnimmt [17]. Wird durch Drogen eine unphysiologisch hohe Dopaminfreisetzung ausgelöst, führt das zur Enkodierung eines sog. Vorhersagefehlers, d. h. die Drogenwirkung ist besser als erwartet und die betroffene Person ist motiviert, den Drogenkonsum zu wiederholen [14, 18, 19].

Beim wiederholten Drogenkonsum kommt es zu einer zunehmenden Gewohnheitsbildung, die im Tiermodell mit der Verschiebung der Aktivierung vom ventralen in Richtung dorsales Striatum verbunden ist [20, 21]. Eine Untersuchung bei Personen mit Alkoholabhängigkeit beobachtete ebenfalls eine Aktivierung im dorsalen gegenüber dem ventralen Striatum bei Entwicklung einer Abhängigkeitserkrankung [22], während dies in anderen Untersuchungen nicht der Fall war [23, 24]. Aktuelle Untersuchungen, die die Gewohnheitsbildung mit komputationaler Modellierung des Verhaltens erfassen wollen, zeigen keine generelle Zunahme habituellen Verhaltens bei Menschen mit Abhängigkeitserkrankungen; vielmehr sind Personen mit Abhängigkeitserkrankungen offenbar dann besonders rückfallgefährdet, wenn zur Tendenz der Gewohnheitsbildung auch eine hohe Erwartung gegenüber den Alkoholwirkungen vorhanden ist [25]. Hinzu kommt eine verstärkte Reaktion auf konditionierte Alkoholreize, wobei hier in bildgebenden Untersuchungen eine verstärkte Aktivierung des medialen präfrontalen Kortex in mehreren Untersuchungen mit einer hohen Rückfallneigung nach Entgiftung verbunden war [23, 26]. Das starke Verlangen nach der abhängigkeitserzeugenden Substanz wird daher vermutlich durch Aktivierung entsprechender motivationaler Netzwerke begleitet oder bedingt, wobei dem ventralen Striatum und weiterer limbischer Regionen eine wesentliche Rolle zugeschrieben wird.

Demgegenüber wird die kognitive Kontrolle über den Drogenkonsum in der Regel als Funktion des präfrontalen Kortex verstanden [5, 8]. Da Striatum und frontaler Kortex allerdings in unterschiedlichsten Regelkreisen verbunden sind, wäre es nicht richtig, hier von eigenständig operierenden Hirnzentren zu sprechen. Es wäre falsch anzunehmen, dass im frontalen Kortex eine Art unabhängige Kontrollinstanz „sitzt“, die durch übermäßige Begierde aus älteren Hirnregionen quasi „überrannt“ wird. Vielmehr sind diese Hirnregionen in engster Interaktion, und bei der Entwicklung von Suchterkrankungen zeigte sich, dass das ventrale Striatum und der präfrontale Kortex gemeinsam und gleichsinnig verstärkt reagieren, wenn eine Personen sich für einen schädlichen Alkoholkonsum entscheidet [27].

Alkohol und andere Drogen haben neurotoxische Wirkungen, die die Funktion des präfrontalen Kortex direkt beeinträchtigen können. Allerdings zeigten Untersuchungen des mit dem frontalen Kortex verbundenen Arbeitsgedächtnisses, dass Menschen mit Alkoholabhängigkeit gegenüber gesunden Kontrollpersonen üblicherweise offenbar kaum Einschränkungen aufweisen [8].

Bezüglich der Aspekte des starken Verlangens nach Alkohol oder einer anderen Substanz und einer verminderten Kontrolle über den Substanzgebrauch handelt es sich neurobiologisch um Aspekte integrierter Regelkreise, die gemeinsam auftreten können, aber auch – insbesondere bei differenzieller neurotoxischer Schädigung – unabhängig voneinander ausgeprägt sein können. In der ICD-11 genügt, dass einer dieser beiden Aspekte erfüllt ist, damit das Kriterium als Hinweis auf das Bestehen einer Abhängigkeitserkrankung gewertet werden kann. Aufgrund der klinischen Überlappung der Symptome und der neurobiologisch verflochtenen Regelkreise erscheint diese Zusammenführung sinnvoll.

Im Bereich dieser Phänomene besteht die stärkste Überlappung zu den sog. Verhaltenssüchten, d. h. der Glückspielsucht und des pathologischen Spielens, die in der ICD-11 gesondert gelistet werden. Wiederum erscheint es sinnvoll, dass das alleinige Vorliegen eines starken Verlangens oder einer Kontrollminderung ohne Toleranzentwicklung oder Entzugssymptomatik einerseits oder die noch zu diskutierenden schädlichen Folgen des Drogenkonsums andererseits nicht ausreichen, um eine Suchterkrankung zu diagnostizieren. Denn starkes Verlangen und Kontrollminderung sind Phänomene, die sich bei jeder leidenschaftlichen Tätigkeit der Menschen nachweisen lassen können und die deshalb per se nicht pathologisch sind [5]. In dem von der WHO bereits veröffentlichten Beschreibungen wird der „internal drive“ besonders betont, der sich nicht nur als subjektiv starkes Verlangen zeigen kann („craving“), sondern auch im Verlust der Kontrolle über den Drogenkonsum, in der zunehmenden Priorität des Drogenkonsums über andere Tätigkeiten und im fortgesetzten Konsum trotz schädlicher oder nachteiliger Auswirkungen. Damit wird der Beobachtung Rechnung getragen, dass ein subjektives Verlangen nach der Droge bei alkoholabhängigen Patienten nicht notwendigerweise auftreten und mit einem erhöhten Rückfallrisiko verbunden sein muss und dass unterschiedliche neurobiologische Korrelate der Kontrollminderung, des Verlangens nach Alkohol und der Vernachlässigung anderer Aktivitäten identifiziert werden konnten [28–30]. Es bleibt abzuwarten, ob sich die von Saunders und Kollegen vorab veröffentlichten Kriterien mit den endgültigen der WHO decken oder ob hier noch Akzentverschiebungen beispielsweise in der Gewichtung des Cravings erfolgen.

## Substanzgebrauch als Priorität im Leben, die trotz schädlicher Folgen persistiert

Das 3. neue Kriterium der Abhängigkeitserkrankungen führt zwei heterogen wirkende Kriterien der ICD-10 zusammen, einerseits die zunehmende Vernachlässigung alternativer Verpflichtungen und Vergnügen zugunsten des Drogenkonsums und andererseits den fortgesetzten Drogenkonsum trotz schädlicher Konsequenzen. Das Kriterium in der ICD-10 „Anhaltender Konsum trotz eindeutiger schädlicher Folgen“ wird in der ICD-11 weniger scharf formuliert und es wird nur noch gefordert, dass der Konsum „oft“ trotz des Auftretens von „Problemen“ fortgesetzt wird.

Klinisch ist es durchaus plausibel, dass zu den Voraussetzungen für die Diagnose einer Substanzgebrauchsstörung schädliche Konsequenzen gehören. Würde das Individuum wegen der Substanzgebrauchsstörung keinerlei nachweisbare Schädigungen erleiden, stellt sich die Frage, warum die Medizin überhaupt zuständig sein sollte. Warum der Aspekt der jetzt recht allgemein betitelten „Probleme“ aber zusammengeführt werden soll mit der zunehmenden Vernachlässigung alternativer Vergnügen, ist nicht wirklich verständlich. Dass die WHO diesen oder einen ähnlich weit auslegbaren Begriff verwenden wird, ist sehr wahrscheinlich, da in der von der WHO bereits veröffentlichten Beschreibung der Alkoholabhängigkeit (Tab. 1) sogar von anhaltendem Konsum trotz negativer Konsequenzen („negative consequences“) gesprochen wird. In diesem Punkt werden offenbar in der ICD-11 unterschiedliche Phänomene vermengt: einerseits die Vernachlässigung anderer Vergnügen, deren neurobiologisches Korrelat im Tiermodell und beim Menschen mit GABAergen Funktionsstörungen im Bereich der Amygdala in Verbindung gebracht werden konnte [28], und andererseits das Auftreten von Problemen wie etwa der finanziellen Verschuldung durch den Kauf von Drogen, deren Preis auch durch gesellschaftliche und gesetzliche Vorgaben beeinflusst wird. Die Zusammenführung dieser unterschiedlichen Aspekte in ein einziges Kriterium überzeugt demnach nicht. Sie ist offenbar der Absicht geschuldet, die bisherigen 6 Kriterien auf 3 zu reduzieren, ohne dass zwingende inhaltliche Gründe für dieses schematisch anmutende Vorgehen ersichtlich werden.

### Infobox 1 Die Probe aufs Exempel: Ist die Atemsucht eine Sucht?

Will man den Sinn von Krankheitsklassifikationen testen, lohnt es sich, imaginäre Beispiele zu überprüfen. Gibt es also beispielsweise eine Atemsucht? Würde die ICD-11-Klassifikation dazu führen, dass das Atmen als Suchterkrankung im Sinne der Substanzgebrauchsstörung klassifizierbar wäre, dann wäre die Definition zu hinterfragen. In der bisherigen ICD-10 ist das bisher nicht der Fall. Zwar kann man davon sprechen, dass es bei Unterbrechung der Luftzufuhr zu starken Entzugserscheinungen und zu einem starken Verlangen nach dem Atmen kommt. Nicht erfüllt sind aber das Kriterium des Schadens durch das Atmen, es gibt keine Toleranzentwicklung in dem Sinne, das im Laufe des Atmens immer größere Mengen an Luft konsumiert werden müssen, um die ursprünglich erwünschte Wirkung zu erzielen oder einer Entzugssymptomatik vorzubeugen, und es gibt keine Kontrollminderung gegenüber dem Atmen (sieht man von anderweitig verursachten Fällen angstbedingter Hyperventilation ab). Auch eine Verengung der Verhaltensvielfalt auf das Atmen findet sich allenfalls als bewusst gewählter Akt bei meditativen Techniken, nicht aber als ungewollte Folge einer Suchterkrankung. Im ICD-10 gibt es also keine Atemsucht.

In der ICD-11 ist diese Frage nicht mehr so einfach zu beantworten, denn hier genügt es ja, dass einer der Aspekte innerhalb der jeweils paarweise neu zusammengeführten Kriterien erfüllt sein muss, um eine Abhängigkeitserkrankung zu diagnostizieren. Das Auftreten von Entzugssymptomen beim plötzlichem Unterbrechen der Luftzufuhr würde also genügen, um eines der 3 Kriterien zu erfüllen, das 2. Kriterium wäre erfüllt, wenn das starke Verlangen zu Atmen und eine reaktive Hyperventilation bei kurzfristiger Unterbrechung der Substanzzufuhr als Kontrollminderung mit Craving gewertet wird. Damit wären aber 2 von 3 Kriterien erfüllt und die Diagnose der „Atemsucht“ könnte gestellt werden. Was hier ironisch klingt, kann aber politisch gefährlich werden: Überträgt man die Suchtkriterien auch auf die Verhaltenssüchte, könnte etwa eine starke persönliche Motivation zum politisch kritischen Bloggen auf Kosten anderer Verpflichtungen unter bestimmten gesellschaftlichen Bedingungen pathologisiert werden.

## ICD-11 in der Praxis

**Figure 1:**
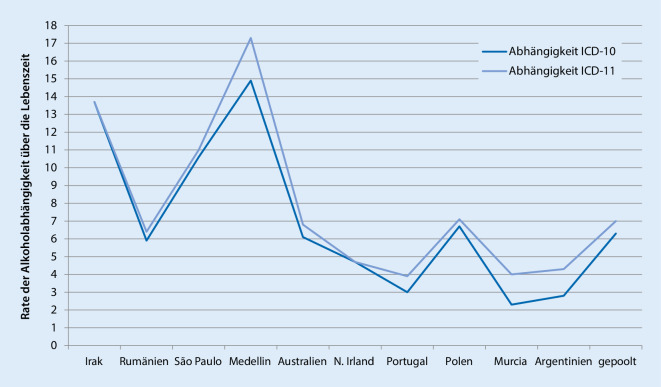

Neben der Frage, ob bisher nicht als Suchterkrankungen verstandene Konsummuster oder Verhaltensweisen als Abhängigkeitserkrankung klassifizierbar werden, stellt sich die Frage, ob die Anwendung der ICD-11-Kriterien die Zahl oder Gruppe der Personen signifikant verändert, die bisher in der ICD-10 beispielsweise als substanzabhängig diagnostiziert wurden. In der bisher größten Studie von Degenhard und Kollegen wurden in 10 Ländern bzw. Regionen in der Welt bei über 12.000 Personen die Konkordanz von der ICD-10, ICD-11 sowie der DSM-IV und DSM‑5 bei Alkohol- und Cannabiskonsum [31] untersucht. Hier zeigte sich, dass in 8 von 10 Regionen und in allen Regionen mit mittleren Einkommen (Brazilien-Sao Paolo, Kolumbien-Medellin und Rumänien) die Alkoholabhängigkeit in der ICD-11 häufiger als in der ICD-10 diagnostiziert wurden (Tab. 3 und Abb. 1; [31]). In einer gepoolten Analyse war die Rate der Alkoholabhängigkeit bei erwachsenen alkoholkonsumierenden Personen unter der Verwendung der ICD-11 bei 7 und damit um 10 % höher als unter Verwendung der ICD-10, bei der die Rate 6,3 betrug [31]. Auch wenn dieser Unterschied in der Studie nicht statistisch signifikant war, ist er klinisch relevant und würde für Deutschland bedeuten, dass anhand der ICD-11 zu den bisher schätzungsweise 2,9 Mio. Menschen mit Alkoholabhängigkeit (entspricht 3,5 % der Bevölkerung [32]) zusätzlich etwa 300.000 weitere Personen die Diagnose einer Alkoholabhängigkeit bekämen.

**Table 3:** 

Definition	Irak	Rumänien	São Paulo	Medellin	Australien	Nordirland	Portugal	Polen	Murcia	Argentinien	Gepoolt	Range
	(*n* = 60)	(*n* = 318)	(*n* = 1480)	(*n* = 399)	(*n* = 5409)	(*n* = 1084)	(*n* = 949)	(*n* = 1333)	(*n* = 424)	(*n* = 726)	(*n* = 12.182)	
Abhängigkeit ICD-10	13,7	5,9	10,6	14,9	6,1	4,7	3	6,7	2,3	2,8	6,3	2,3–14,9
Abhängigkeit, ICD-11	13,7	6,4	11	17,3	6,8	4,7	3,9	7,1	4	4,3	7	3,9–17,3

Eine weitere Arbeit zeigt bei Jugendlichen sogar eine 2,3-fach höhere Prävalenz der Alkoholabhängigkeit im ICD-11 gegenüber der Verwendung des ICD-10 [33] und etwa 50 % häufiger die Diagnose der Cannabisabhängigkeit unter Verwendung des ICD-11. Dies sind klinisch relevante Unterschiede und weisen darauf hin, dass es durch die ICD-11 zu einer Zunahme der Abhängigkeitsdiagnosen kommen kann.

In einer weiteren Veröffentlichung wurde die australische Subgruppe der multinationalen ersten Studie genauer beschrieben. Es zeigten sich keine statistisch signifikanten Unterschiede hinsichtlich des Vorkommens von Alkohol- und Cannabisabhängigkeit zwischen der Klassifikation nach ICD-11, ICD-10 und DSM-IV [34]. Allerdings wurde die Alkoholabhängigkeit mittels ICD-11 insgesamt trotzdem 10 % häufiger als mittels dem ICD-10 diagnostiziert. Auch zeigte der Vergleich der genannten Diagnosesysteme (ICD-10 und ICD-11, DSM-IV) mit der Kategorie der neuen Substanzgebrauchsstörung des DSM-5 nur eine geringe Überschneidung, was Zweifel an der Kategorisierung mittels des DSM‑5 weckt. Aber auch kulturelle und regionale Einflüsse auf die Kategorisierung mittels ICD-11 müssen erwogen werden. Da in den öffentlich zugänglichen Versionen des ICD-11 nur noch von „Problemen“ [1] bzw. „negativen Konsequenzen“ [2] beim Substanzkonsum die Rede ist, können auch soziale Schwierigkeiten darunter gefasst werden, die allein aufgrund regional unterschiedlicher Gesetze eintreten. So kann fortgesetzter Konsum trotz gesetzlichen Verbots zu „Problemen“ oder „negativen Konsequenzen“ ohne eigentlichen Krankheitswert führen, wenn diese Probleme einfach nur im Rahmen der gesetzlichen Strafen und der damit verbundenen persönlichen Nachteile auftreten. Bezüglich der Umsetzung der neuen ICD-11 in die alltägliche Praxis sollte also systematisch untersucht werden, in welchen sozialen Kontexten für die Betroffenen unangemessene Pathologisierung oder Stigmatisierung auftreten könnten.

## Fazit für die Praxis

- Die Änderungen in den Abhängigkeitskriterien der ICD(International Classification of Diseases)-11 umfassen eine Zusammenführung der bisherigen 6 Kriterien der ICD-10 zu 3 Doppelkriterien, die jeweils zwei Aspekte umfassen. Pro Paar muss ein Aspekt bzw. Symptom erfüllt sein.
- Zukünftig müssen nur 2 der 3 Kriterien erfüllt sein.
- Die sozialen Auswirkungen der ICD-11-Neuerungen für Betroffene sollten zukünftig systematisch erforscht werden.
